# Protective Effects of Aqueous Extract of *Baillonella toxisperma* Stem Bark on Dexamethasone-Induced Insulin Resistance in Rats

**DOI:** 10.1155/2019/8075163

**Published:** 2019-08-27

**Authors:** Marius Trésor Wego, Sylviane Laure Poualeu Kamani, David Miaffo, Moise Legentil Nchouwet, Albert Kamanyi, Sylvie Léa Wansi Ngnokam

**Affiliations:** ^1^Department of Animal Biology, Faculty of Science, University of Dschang, P. O. Box 67 Dschang, Dschang, Cameroon; ^2^Department of Life and Earth Sciences, Higher Teachers' Training College, University of Maroua, P. O. Box 55 Maroua, Maroua, Cameroon

## Abstract

The aim of this study was to evaluate the effects of the aqueous extract of *Baillonella toxisperma* stem bark on dexamethasone-induced insulin resistance in rats. A quantitative phytochemical study was done on the aqueous extract of *Baillonella toxisperma* for the total phenol, flavonoid, and flavonol determination. Insulin resistance was induced by intraperitoneal injection of dexamethasone (1 mg/kg) for 8 days, one hour before oral administration of different treatments (extract at doses of 60, 120, and 240 mg/kg and metformin at 40 mg/kg). During the test, body weight and blood glucose level were evaluated on days 1 and 8. At the last day of treatment, the glucose tolerance test was performed in rats; after that, blood samples were collected for triglycerides, total cholesterol, LDL and HDL cholesterols, transaminases (ALT and AST), and total protein level determination. Organs (heart, liver, pancreas, and kidneys) were also collected for the relative organ weight determination. The results showed that the aqueous extract of *B. toxisperma* is rich in total phenols, flavonoids, and flavonols. This extract significantly reversed the metabolic alterations (lipid profile, protein level, and transaminase activity) induced by dexamethasone in rats. At doses of 120 and 60 mg/kg, *Baillonella toxisperma* also significantly decreases (*p* < 0.05; *p* < 0.01) postprandial hyperglycemia in insulin resistance rats. The results suggest that *Baillonella toxisperma* can manage insulin resistance and may be useful for the treatment of type 2 diabetes mellitus.

## 1. Introduction

Diabetes mellitus (DM) refers to a group of chronic metabolic diseases which are generally characterized by hyperglycemia, which eventually leads to damage of multiple body systems [1]. It belongs to the group of five leading important diseases causing death globally and remains a major health problem in Africa [2]. There are two main types of DM: type 1 (T1DM) and type 2 (T2DM) diabetes mellitus. T1DM is due to an impaired insulin production, while T2DM is caused by a combination of genetic and environmental factors, which lead to insulin resistance and impaired insulin secretion. T2DM is the most common form of DM. It is characterized by hyperglycemia and alterations of carbohydrate, protein, and lipid metabolism, caused by a defect in insulin production or its action. The number of people with T2DM is increasing in every country, and it is estimated that 439 million people would have T2DM by the year 2030 [3]. Many progresses in the management of diabetes mellitus using synthetic drugs have been made, but these drugs have many adverse effects. In addition, these drugs are often inaccessible to many local populations because of the difficulties in their distribution. These populations generally use plants for primary health care. Thus, traditional phytotherapeutic approaches seem to enhance an interesting potential whose development process, through appropriate scientific procedures, could offer a credible alternative to communities.

*Baillonella toxisperma*, belongs to the family Sapotaceae, is a big tree widely distributed throughout Cameroon, mostly in Douala and Yabassi [4, 5]. It is traditionally called the “moabi.” It is a large tree that dominates the dense humid forest. It can exceed 60 m in height. Its very straight and cylindrical bole has no shoulder, and its bark is cracked. The elongated leaves have many secondary veins and are grouped at the ends of the branches. The moabi fruit is a drupe sometimes containing several seeds [6]. The maceration of stem barks of *Baillonella toxisperma* is used locally in the management of diabetes mellitus [5], but there is no scientific evidence to support this claim. This study aims to scientifically validate the protective effects of *Baillonella toxisperma* stem bark's aqueous extract on dexamethasone-induced insulin resistance in rats.

## 2. Materials and Methods

### 2.1. Chemicals

All the reagents were obtained commercially. Dexamethasone sodium phosphate, Folin–Ciocalteu reagent, sodium carbonate, sodium nitrite, aluminium chloride, sodium hydroxide, sodium acetate, gallic acid, quercetin, and catechin were purchased from Sigma-Aldrich, St. Louis, USA. d-glucose was purchased from Edu-Lab Biology Kit, Bexwell, Norfolk PE389GA, UK. Diagnostic kits used for estimation of total cholesterol, triglycerides, high density lipoproteins (HDL), low density lipoproteins (LDL), total protein, alanine aminotransferase (ALAT), and aspartate aminotransferase were procured from Inmesco, innovation for medical lab, L-S 04/2009 Germany. All chemicals and reagents were of analytical grade.

### 2.2. Collection of Plant Material

*Baillonella toxisperma* stem barks were collected in Mbalmayo (Central Cameroon) in May 2014. The plant was authenticated in National Herbarium of Cameroon by comparison to the number 6608/HNC. The stem barks were shade-dried, then powdered with a mechanical grinder, passing through a sieve, and stored in an air-tight container.

### 2.3. Preparation of Aqueous Extract

Powdered stem barks (150 g) of *B. toxisperma* were macerated in 1.5 L of distilled water for 48 h at room temperature. The mixture was then filtered through a filter paper, and the filtrate was left for fermentation for 24 h according to the recommendation of the tradipratician. After that, the filtrate was concentrated by evaporating water at 40°C in a drying oven. The percentage yield of the extract was 11.21%.

### 2.4. Phytochemical Analysis

#### 2.4.1. Determination of Total Phenols

Total phenolic content in the extracts was determined by the modified Folin–Ciocalteu method [7]. The reaction mixture consisted of 200 *μ*l of extract, 200 *μ*l·ml of 2N Folin–Ciocalteu reagent, and 400 *μ*l of 20% sodium carbonate solution. The mixture was stirred and incubated in a water bath at 40°C for 20 minutes. The experiment was carried in triplicate. The absorbance was read at 760 nm. Total phenolic content was expressed as mg/g gallic acid equivalent (mg EAG/gE) using the equation obtained from a calibration curve of gallic acid (*y*=0.0213*x*+0.0065; *R*^2^=0.9958).

#### 2.4.2. Determination of Total Flavonoids

Total flavonoids were estimated using the aluminium colorimetric method by Padmaja et al. [8]. In brief, 1500 *μ*l of distilled water and 30 *μ*l of sodium nitrite at 5% were added to 100 *μ*l of the extract. After 5 min of incubation at room temperature, 30 *μ*l of aluminium chloride (10%) and 200 *μ*l of sodium hydroxide (1M) were added to the mixture. The experiment was carried in triplicate. The absorbance was measured at 510 nm. Total flavonoid content was calculated as catechin equivalent (mg EC/gE) using the equation obtained from the calibration curve (*y*=0.2207*x* − 0.0118; *R*^2^=0.9628).

#### 2.4.3. Determination of Flavonol Content

Flavonol content was determined according to the method of Almaraz-Abarca et al. [9]. In test tubes containing 1280 *μ*l of distilled water, 40 *μ*l of extract (2 mg/ml), 40 *μ*l of aluminium chloride (20%), and 40 *μ*l of sodium acetate (5%) were added. The experiment was carried in triplicate. The absorbance was measured after 30 minutes at 415 nm. The flavonol content was calculated as quercetin equivalent (mg EQ/gE) using the equation obtained from the calibration curve (*y*=0.1872*x*; *R*^2^=0.9734).

### 2.5. Experimental Animals

Wistar albino rats of both sexes (180–250 g) were obtained from the animal house of the Department of Animal Biology, Faculty of Science, University of Dschang, Cameroon. The animals were housed at room temperature (22–28°C), in natural luminosity and given standard laboratory feed and water ad libitum. All experiments were conducted in compliance with ethical guide for care and use of laboratory animals. The animals were treated in accordance with the internationally accepted standard ethical guidelines for laboratory animal use and care as described in European Community Guidelines [10].

### 2.6. Oral Glucose Tolerance Test

For this test, 30 overnight fasted (14 h) rats were used and divided into five groups of six animals each. Group 1 received distilled water, group 2 received metformin (40 mg/kg body weight), and groups 3, 4, and 5 were treated with the aqueous extract of *Baillonella toxisperma* (AEBT) at respective doses of 60, 120, and 240 mg/kg of body weight. One hour after administration of different treatments, D-glucose (3 g/kg body weight) was orally administrated to all the rats. Blood glucose was estimated using the ACCU-CHEK Active Glucometer in the blood collected at the tail vein. Blood glucose was recorded before the administration of different substances and at 30, 60, 90, and 120 min after D-glucose treatment.

### 2.7. Dexamethasone-Induced Insulin Resistance

Insulin resistance was induced by intraperitoneal injection of dexamethasone (1 mg/kg) for 8 days. Thirty-six (36) rats were weighed before treatment and then were divided into six groups of six animals each. Animals were fasted overnight (14 h) before dexamethasone treatment as described by Mahendran and Devi [11]. Group 1 served as normal control and received per os (*p.o.*) distilled water and intraperitoneal injection of NaCl (0.9%); group 2 served as insulin resistant control and received distilled water *p.o.* and intraperitoneal injection of dexamethasone; group 3 served as positive control and received metformin (40 mg/kg, *p.o.*) and dexamethasone injection; groups 4, 5, and 6 were treated with AEBT at respective doses of 60, 120, and 240 mg/kg, *p.o.* plus dexamethasone injection. Blood glucose and body weight were evaluated the first and last day of the treatment. At the end of the treatment, the glucose tolerance test was performed in rats.

### 2.8. Biochemical Analysis and Organs' Weight Determination

On the 9th day, the animals were anesthetized and blood was collected under anticoagulant (heparin). Thereafter, plasma was separated for the estimation of lipid profile, transaminases (AST and ALT), and total protein levels using commercial standard diagnostic kits. Immediately after blood collection, organs (heart, liver, pancreas, and kidneys) were removed and weighed for relative organs' weight determination.

### 2.9. Statistics

All the results were expressed as mean ± SEM (standard error of mean). Data were analyzed using one-way ANOVA followed by the Tukey posttest (ALT, AST, lipid, and protein levels) and two-way ANOVA followed by the Bonferroni posttest (blood glucose variation and body weight) using Graph Pad Prism version 5.03 and SPSS logical for phytochemical analysis. *p* < 0.05 was considered significant.

## 3. Results

### 3.1. Quantitative Phytochemical Test

The phytochemical analysis revealed that the aqueous extract of *B. toxisperma* contains a significant amount of phenols, flavonoids, and flavonols estimated at 23.09 ± 1.92 mg EAG/gE, 0.19 ± 0.02 mg EC/gE, and 0.37 ± 0.06 mg EQ/gE, respectively.

### 3.2. Effect of AEBT on the Oral Glucose Tolerance Test in Normal Rats

**Figure 1:**
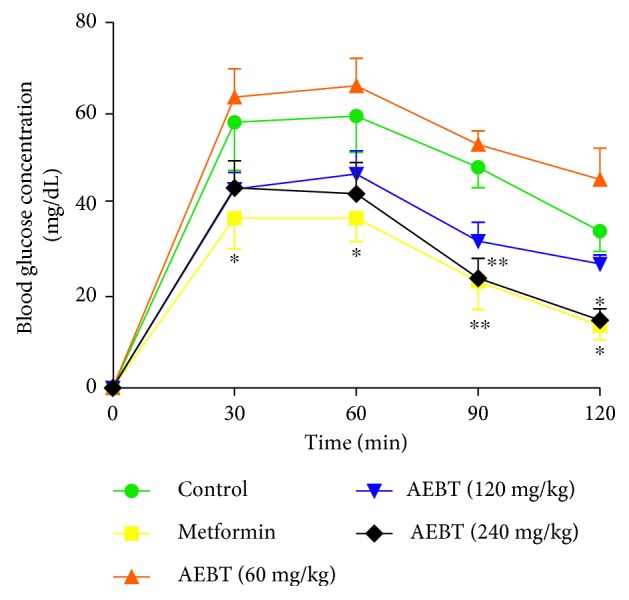
Effect of the aqueous extract of *Baillonella toxisperma* on hyperglycemia induced by D-glucose in normoglycemic rats. AEBT: aqueous extract of *B. toxisperma*. ^*∗*^*p* < 0.05; ^*∗∗*^*p* < 0.01 compared to the control group. *n*=6; data are presented as mean ± SEM.

Administration of AEBT resulted in a significant reduction of postprandial blood glucose concentration. The best effect was observed at dose of 240 mg/kg with a significant reduction of 50.34% from the 90^th^ min (*p* < 0.05) which was emphasized after 120 min (69.31%; *p* < 0.01) (Figure 1).

### 3.3. Effects of AEBT on Dexamethasone-Induced Insulin Resistance in Rats

#### 3.3.1. Effect on Body Weight

**Figure 2:**
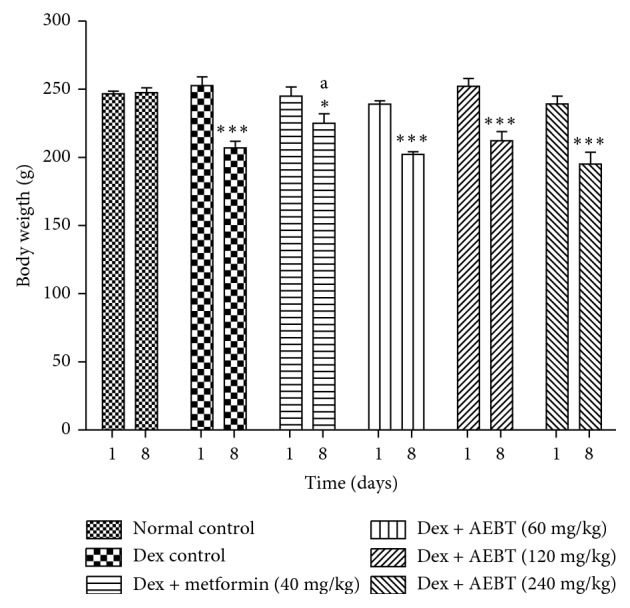
Effect of the aqueous extract of *Baillonella toxisperma* on body weight of insulin resistant rats. Dex: dexamethasone; AEBT: aqueous extract of *B. toxisperma*. ^*∗∗∗*^*p* < 0.001 compared to the normal control group; ^a^*p* < 0.05 compared to the dexamethasone control group. *n*=6; data are presented as mean ± SEM.

Dexamethasone significantly reduced (*p* < 0.001) the body weight of rats after 8 days of administration, except on metformin-treated rats. Treatment with the aqueous extract of *B. toxisperma* did not prevent this weight loss (Figure 2).

#### 3.3.2. Effect of AEBT on the Blood Glucose Level

**Figure 3:**
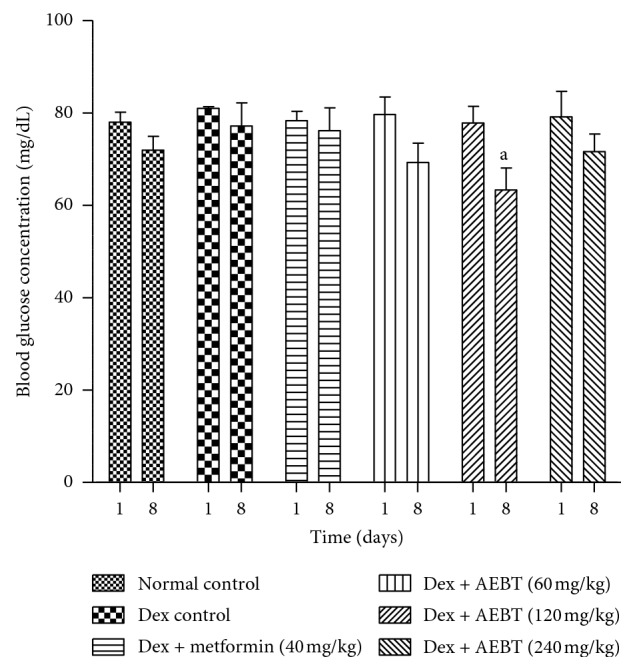
Effect of the aqueous extract of *Baillonella toxisperma* on the blood glucose level in dexamethasone-induced insulin resistant rats. Dex: dexamethasone; AEBT: aqueous extract of *B. toxisperma*; ^a^*p* < 0.05 compared to the dexamethasone control group. *n*=6; data are presented as mean ± SEM.

There was no significant variation of the blood glucose level of rats after 8 days of dexamethasone administration. However, extract at dose of 120 mg/kg significantly reduced (*p* < 0.05) the blood glucose level of rats by 29.62% compared to the dexamethasone control group (Figure 3).

### 3.4. Effect of AEBT on the Glucose Tolerance Test in Dexamethasone-Treated Rats

**Figure 4:**
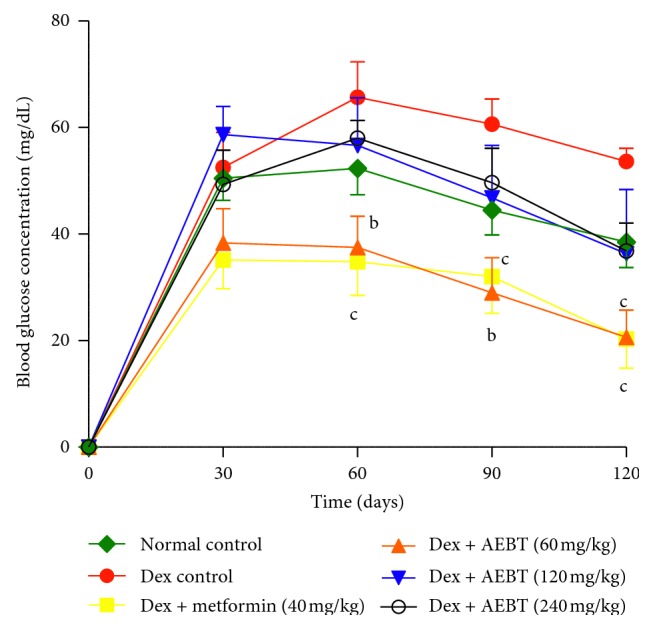
Effect of the aqueous extract of *Baillonella toxisperma* on the glucose tolerance test in dexamethasone-treated rats. Dex: dexamethasone; AEBT: aqueous extract of *B. toxisperma*; ^b^*p* < 0.01; ^c^*p* < 0.01 compared to the dexamethasone control group. *n*=6; data are presented as mean ± SEM.

Metformin (40 mg/kg) and AEBT at dose of 60 mg/kg significantly reduced (*p* < 0.01; *p* < 0.001) the postprandial hyperglycemia in dexamethasone rats from the 60^th^ to the 120^th^ min (Figure 4).

### 3.5. Effect of AEBT on Biochemical Parameters

Effects of *B. toxisperma* on biochemical parameters of insulin-resistant rats are presented in Table 1. From this table, it appears that 8 days of dexamethasone injection significantly decreased (*p* < 0.05) the total protein level of dexamethasone control rats compared to normal control rats. AEBT and metformin protected the rats against this reduction of plasma protein levels.

As shown in the same table, dexamethasone administration (1 mg/kg) significantly increased the levels of triglycerides (*p* < 0.01), total cholesterol, and LDL cholesterols (*p* < 0.001) and decreased (*p* < 0.001) the level of HDL cholesterol. However, metformin and the extract attempted to reverse this situation notably as regards the level of plasma triglycerides.

ASAT and ALAT activity significantly increased (*p* < 0.001) in dexamethasone control rats compared to normal control rats. Treatment with AEBT (120 and 240 mg/kg) significantly decreases ASAT and ALAT activity.

### 3.6. Effect of AEBT on Relative Organ Weight

**Figure 5:**
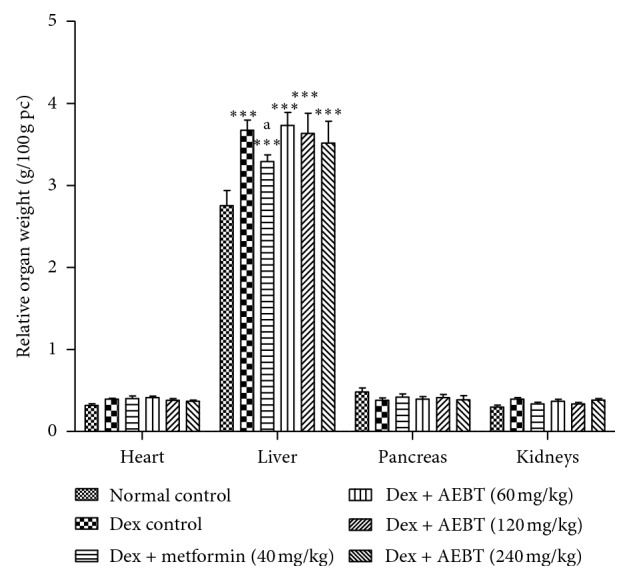
Effect of the aqueous extract of *Baillonella toxisperma* on relative organ weight of insulin-resistant rats. Dex: dexamethasone; AEBT: aqueous extract of *B. toxisperma*. ^*∗∗∗*^*p* < 0.001 compared to the normal control group; ^a^*p* < 0.05 compared to the dexamethasone control group. *n*=6; data are presented as mean ± SEM.

Dexamethasone induced a significant increase (*p* < 0.001) of liver weight of rats compared to the normal control group but did not affect the other organ's weight (heart, kidneys, and pancreas). Only the metformin (40 mg/kg) treatment significantly reduced (*p* < 0.05) the liver weight gain (Figure 5).

## 4. Discussion

Dexamethasone is a synthetic glucocorticoid whose chronic exposure to high doses causes insulin resistance [12]. In the present study, experimental induction of insulin resistance by dexamethasone at dose of 1 mg/kg administered continuously for 8 days led to profound alterations of metabolic parameters characterized by high levels of total cholesterol, LDL cholesterol, and triglyceride and low levels of HDL cholesterol, total protein, and body weight in rats.

Insulin resistance promotes the increase of hormone-sensitive lipase activity of adipose tissue and the decrease of lipoprotein lipase activity. This leads to an increase in fatty acids mobilization from adipocytes and an increase in hepatic synthesis of triglycerides, which are released into the bloodstream as VLDL cholesterol [13]. According to Halimi [14], the decrease in the plasma concentration of HDL cholesterol can be explained by the presence in the circulating blood of a particular enzyme, the “Cholesterol Ester Transfer Protein” (CETP). Under hypertriglyceridemia conditions, this enzyme transfers triglycerides from VLDL cholesterol to HDL cholesterol. HDL cholesterols thus enriched with triglycerides are rapidly hydrolysed, and because of their increased catabolism, the blood level of HDL decreases. Hypercholesterolemia could be due to an increase of triglycerides in the liver, which would be responsible for an increase of the “Sterol Regulatory Element-Binding Protein”49 (SREBP) expression, a factor regulating the capture and cholesterol synthesis [13]. In this study, the various treatments administered significantly reduced triglycerides, total cholesterol, and LDL cholesterol and increased HDL cholesterol levels. These results could thus reflect the ability of the aqueous extract of *B. toxisperma* to improve the tissue sensitivity to insulin, thus reducing the hormone-sensitive lipase activity and increasing the lipoprotein lipase activity, resulting in a decrease of lipolysis. Aqueous and methanol extracts of *B. toxisperma* are therefore endowed with hypolipidemic properties. Flavonoids have been shown to improve dyslipidemia [15–17]. Thus, the hypolipidemic effect of *B. toxisperma* could be attributed to the flavonoids contained in the plant. These extracts could therefore effectively prevent cardiovascular complications related to diabetic dyslipidemia.

AST and ALT are markers of liver function whose release into the bloodstream results from the loss of hepatocyte membrane integrity and cell damage [18]. It is well known that glucocorticoids increase lipid catabolism. This increase of lipolysis results in a massive release of fatty acids which are accumulated in the liver [19]. This fatty acid accumulation in the liver causes hepatic steatosis, accompanied by inflammation, leading to an increase in liver mass and long-term tissue necrosis [20]. Thus, the significant increase in ALT and AST activities and relative liver weight in dexamethasone rats in this study would be due to the toxic effects of dexamethasone on the liver. The decrease in transaminase levels in animals treated with the aqueous extract indicates that *B. toxisperma* could have the ability to reverse the hepatotoxic effects of dexamethasone on the liver. This corroborates the results obtained by Bomgning et al. [21] who demonstrated the hepatoprotective properties of the aqueous and methanol extracts of *B. toxisperma*. This hepatoprotective effect could be related to polyphenolic compounds present in the plant and whose hepatoprotective effects have been demonstrated [22, 23].

In the present study, the aqueous extract of *B. toxisperma* protected the rats against the significant reduction of total protein levels induced by dexamethasone. This effect would reflect an insulin sensitizing effect of these extracts and, consequently, a decrease in proteolysis. The decrease in body weight and relative liver weight of rats observed was not affected by the extract.

From the results of this study, dexamethasone did not modify significantly the blood glucose of rats. However, in animals treated with the aqueous extract at dose of 120 mg/kg, there was a significant decrease in blood glucose. These results suggest that *B. toxisperma* extracts may also act by stimulating insulin secretion and thus have hypoglycemic effects. These hypoglycemic effects could be related to the presence of flavonoids and flavonols in the plant extract. In fact, N'diaye et al. [24] showed that these compounds possess antidiabetic properties. In normal and dexamethasone-treated rats, the aqueous extract of *B. toxisperma* was effective in preventing the impaired glucose tolerance. These observations suggest that *B. toxisperma* would be able to prevent dexamethasone induce insulin resistance.

## 5. Conclusion

In conclusion, our investigations indicate that the aqueous extract of *B.* toxisperma has demonstrated hypoglycemic, hypolipidemic, and hepatoprotective effects on dexamethasone-induced insulin resistance in rats. These effects could be due to the presence of phenols and flavonoids present in the plant.

## Figures and Tables

**Table 1:** Effect of *Baillonella toxisperma* on biochemical parameters in dexamethasone-induced insulin resistance in rats.

	Total proteins (g/dL)	Total cholesterol (mg/dL)	Triglycerides (mg/dL)	HDL cholesterol (mg/dL)	LDL cholesterol (mg/dL)	ALT (U/L)	AST (U/L)
Normal control	6.52 ± 0.42	58.85 ± 4.93	51.25 ± 7.51	33.08 ± 2.946	15.52 ± 6.472	39.99 ± 5.31	30.19 ± 2.49
Dex control	4.78 ± 0.29^*∗*^	97.94 ± 2.60^*∗∗∗*^	85.71 ± 2.52^*∗∗*^	13.30 ± 0.59^*∗∗∗*^	67.50 ± 2.55^*∗∗∗*^	78.81 ± 4.19^*∗∗∗*^	112.5 ± 5.60^*∗∗∗*^
Dex + Metf (40 mg/kg)	5.26 ± 0.54	70.60 ± 4.07^c^	49.64 ± 6.03^c^	17.22 ± 0.36^*∗∗∗*^	43.46 ± 3.75^*∗∗*b^	54.19 ± 5.39	43.94 ± 5.14^c^
Dex + AEBT (60 mg/kg)	5.12 ± 0.20	70.45 ± 4.37^c^	65.71 ± 5.29	19.67 ± 2.02^*∗∗∗*^	37.63 ± 4.81^*∗*c^	119.7 ± 5.80^*∗∗∗*c^	120.3 ± 12.72^*∗∗∗*^
Dex + AEBT (120 mg/kg)	5.86 ± 0.45	88.76 ± 3.07^*∗∗∗*^	46.32 ± 5.32^c^	21.84 ± 1.98^*∗∗*a^	57.65 ± 4.26^*∗∗∗*^	62.22 ± 6.71	79.29 ± 9.37^*∗∗∗*a^
Dex + AEBT (240 mg/kg)	5.72 ± 0.39	91.93 ± 3.35^*∗∗∗*^	49.45 ± 5.28^c^	24.87 ± 2.53^b^	57.18 ± 3.60^*∗∗∗*^	17.34 ± 6.81^c^	73.30 ± 3.53^*∗∗*b^^b^

Dex: dexamethasone; AEBT: aqueous extract of *B. toxisperma*; Metf: metformin. ^*∗*^*p* < 0.05, ^*∗*^*p* < 0.01, and ^*∗∗∗*^*p* < 0.001 compared to the normal control group; ^a^*p* < 0.05, ^b^*p* < 0.01, and ^c^*p* < 0.001 compared to the dexamethasone control group. *n*=6; data are presented as mean ± SEM.

## Data Availability

The data that support the findings of this study are available from the corresponding author upon reasonable request.
